# Targeting stemness is an effective strategy to control *EML4-ALK*^+^ non-small cell lung cancer cells

**DOI:** 10.18632/oncotarget.5434

**Published:** 2015-10-22

**Authors:** Se Jin Oh, Kyung Hee Noh, Young-Ho Lee, Soon-Oh Hong, Kwon-Ho Song, Hyo-Jung Lee, Soyeon Kim, Tae Min Kim, Ju-Hong Jeon, Jae Hong Seo, Dong-Wan Kim, Tae Woo Kim

**Affiliations:** ^1^ Laboratory of Infection and Immunology, Graduate School of Medicine, Korea University, Seoul, Korea; ^2^ Department of Biochemistry & Molecular Biology, College of Medicine, Korea University, Seoul, Korea; ^3^ Department of Internal Medicine, Seoul National University College of Medicine, Seoul, South Korea; ^4^ Department of Physiology, Seoul National University, College of Medicine, Seoul, South Korea; ^5^ Division of Oncology, Department of Internal Medicine, College of Medicine, Korea University, Seoul, Republic of Korea

**Keywords:** EML4-ALK, stemness factor, rapamycin, resistance, NSCLC

## Abstract

The fusion between anaplastic lymphoma kinase (*ALK*) and echinoderm microtubule-associated protein-like 4 (*EML4*) is a causative factor in a unique subset of patients with non-small cell lung carcinoma (NSCLC). Although the inhibitor crizotinib, as it blocks the kinase activity of the resulting EML4-ALK fusion protein, displays remarkable initial responses, a fraction of NSCLC cases eventually become resistant to crizotinib by acquiring mutations in the *ALK* domain or activating bypass pathways via EGFR, KIT, or KRAS. Cancer stem cell (CSC) theory provides a plausible explanation for acquisition of tumorigenesis and resistance. However, the question as to whether EML4-ALK-driven tumorigenesis is linked with the stem-like property and whether the stemness is an effective target in controlling EML4-ALK^+^ NSCLC including crizotinib-resistant NSCLC cells has not been addressed. Here, we report that stem-like properties stem from ALK activity in EML4-ALK^+^ NSCLC cells. Notably, treatment with rapamycin, a CSC targeting agent, attenuates stem-like phenotypes of the EML4-ALK^+^ cells, which increased capability of tumor formation and higher expression of stemness-associated molecules such as ALDH, NANOG, and OCT4. Importantly, combinational treatment with rapamycin and crizotinib leads to synergistic anti-tumor effects on EML4-ALK^+^ NSCLC cells as well as on those resistant to crizotinib. Thus, we provide a proof of principle that targeting stemness would be a novel strategy to control intractable *EML4-ALK^+^* NSCLC.

## INTRODUCTION

About 3%–7% of NSCLC tumors are driven by an activating fusion of anaplastic lymphoma kinase (*ALK*) and echinoderm microtubule-associated protein-like 4 (*EML4*) genes [1, 2]. The resulting EML4-ALK fusion protein is constitutively active in these cancer cells and is a driver of tumorigenesis for these cells. In this fusion protein, the complete intracellular portion of *ALK* is preserved, and hence cancer cells harboring this fusion are sensitive to ALK tyrosine kinase inhibition [3]. Crizotinib was approved as a first-in-class ALK inhibitor for the treatment of EML4-ALK*^+^* NSCLC patients. Although most patients with *ALK*-rearranged NSCLC derive substantial clinical benefit from crizotinib, a fraction of tumors eventually become resistant to crizotinib [4]. The acquired resistance to crizotinib is highly associated with secondary mutations in the ALK tyrosine kinase domain and amplification of the *ALK* fusion gene. Among them, L1196M in the *ALK* gatekeeper site was first identified in a crizotinib-resistant patient in Japan [5]. Also, non-gatekeeper mutations such as L1152R, C1156Y, and G1269A, were proposed to be associated with resistance to inhibitors used in the treatment of *ALK*^+^ NSCLC [5–7]. In addition, another mechanism of *ALK*-dependent resistance occurs upon activation of alternative signaling pathways via EGFR, KIT, or KRAS with known tumor promoting properties [8–11]. Hence, considerable efforts are now underway to develop second-generation ALK inhibitors designated to overcome this clinical challenge. Unfortunately, however, crizotinib-resistant NSCLC becomes resistant to the second-generation inhibitors by acquiring additional mutations in the *ALK* domain or activating the bypass signaling pathways. Thus, there is an urgent need to clinically develop a novel and fundamental strategy which can break the vicious cycle of acquired *ALK* resistance.

In the cancer stem cell (CSC) hypothesis, CSCs denote a subtype of cancer cells that has the ability to self-renew and generate diversity of cell in the tumor [12, 13]. These cells have been characterized with ‘stem-like’ properties and although may be few in number, they may be drivers of tumorigenesis in a tumor bulk [14, 15]. In spite of controversies in the cancer stem cell theory, there have been many reports about the existence of a small population of stem-like cancer cells in multiple types of human cancer including NSCLC [16–18]. It is notable that the stem-like property of CSCs may be linked with intractable tumor recurrence and a causative reason for therapeutic failure [15, 19, 20]. Furthermore, it has recently been shown that the CSC-targeting drugs used to treat recurrent and intractable cancer provide superior benefit in cancer treatment to conventional cancer drugs, although their exact mechanism of action remains to be determined [21–23].

Here, we report that EML4-ALK-driven tumorigenesis is linked with a stem-like property and that the ALK activity plays a key role in maintaining stem-like properties of EML4-ALK^+^ NSCLC cells as characterized by increased capability of tumor formation and expression of stemness-associated molecules such as ALDH, NANOG, and OCT4. Notably, we demonstrate that rapamycin, a CSC-specific target, is effective in reversing the stem-like properties of the EML4-ALK^+^ cells. Moreover, the combinational treatment with rapamycin and crizotinib leads to synergistic anti-tumor effects on EML4-ALK^+^ NSCLC cells as well as on those that acquired resistance to crizotinib. Taken together, our findings show that CSC drugs targeting stem-like traits of cancer cells could be effective in controlling refractory EML4-ALK^+^ NSCLC.

## RESULTS

### *EML4-ALK* increases stem-like properties of NSCLC cells *in vitro* and *in vivo*

**Figure 1:**
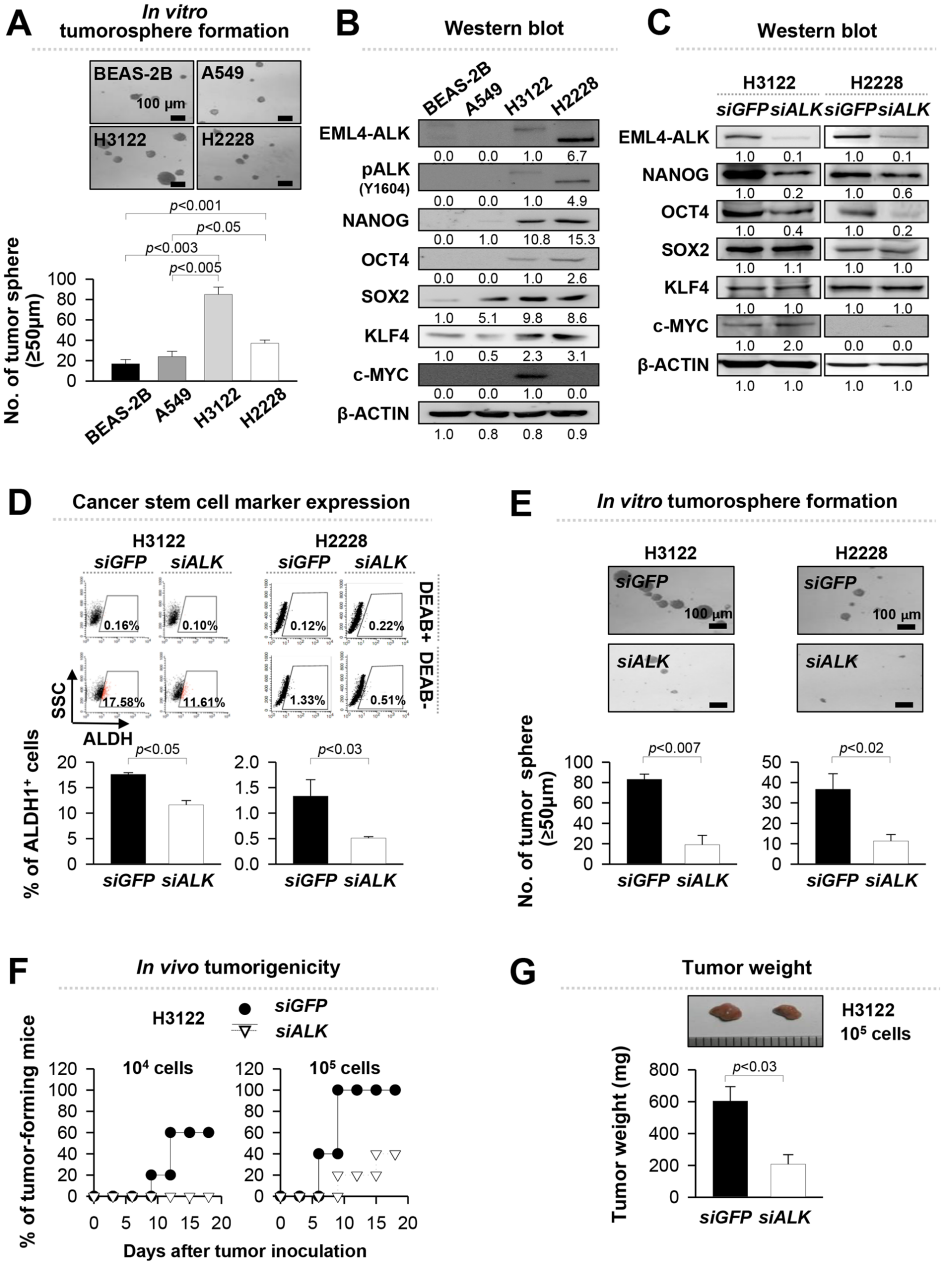
*EML4-ALK* increases the stem-like properties and tumorigenicity of *EML4-ALK*-driven NSCLC cells *in vitro* and *in vivo* **A.** Sphere-forming capacity of BEAS-2B, A549, H3122 and H2228 cells in a low-density suspension culture. Original magnification, x40. **B.** ALK, pALK, NANOG, OCT4, SOX2, KLF4, c-MYC and β-ACTIN expression in BEAS-2B, A549, H3122 and H2228 cells in a low-density suspension culture. Original magnification, x40. (B) ALK, pALK, NANOG, OCT4, SOX2, KLF4, c-MYC and β-ACTIN expression in BEAS-2B, A549, H3122 and H2228 cells was visualized by western blot analysis with lysates from the monolayer cultured cells. (C) H3122 and H2228 cells were treated with *siGFP* (control) or *siALK* and the levels of ALK, NANOG, OCT4, SOX2, KLF4, and c-MYC proteins were analyzed. β-ACTIN was used as an internal loading control. Numbers below blots indicate expression as measured by fold change. **D.** Flow cytometry analysis of the frequency of ALDH1^+^ cells in H3122 and H2228 cells treated with *siALK* or *siGFP* (control). **E.** Sphere-forming capacity of H3122 and H2228 cells treated with *siGFP* or *siALK* in a low-density suspension culture. Original magnification, × 40. **F.** Tumorigenicity of *siGFP*-versus *siALK*-treated H3122 cells inoculated at indicated doses into 5 NOD/SCID mice per group. **G.** Tumors were extracted at 20 days after injection of 10^5^ *siGFP*- or *siALK*-treated H3122 cells. Error bars represent mean ± SD. Individual data analysis was performed using two-tailed Student's *t*-test.

Previous studies have reported that stem-like properties are mechanistically linked with tumorigenicity of cancer [16–18]. To identify the role of *EML4-ALK* fusion oncogene in stem-like properties of NSCLC cells, we performed a tumorosphere-forming assay using four different lung epithelial cell lines, including primary and immortalized human bronchial epithelial cells (BEAS-2B), EML4-ALK*^−^* NSCLC cells (A549), and EML4-ALK^+^ NSCLC cells (*EML4-ALK* variant 1 (*EAV1*)-expressing H3122 and *EML4-ALK* variant 3 (*EAV3*)-expressing H2228). As shown in Figure 1A, EML4-ALK^+^ cells (H3122, H2228) had higher tumorosphere-forming capacity with statistical significance than immortalized primary epithelial cells (BEAS-2B) and EML4-ALK^−^ NSCLC cells (A549) when cultured under suspension conditions within minimal growth factor supplementation. To assess the molecular basis for the enhanced stem-like phenotype of EML4-ALK^+^ cells, we also examined the expression of the stem cell related molecules such as NANOG, OCT4, SOX2, KLF4, and c-MYC and the activated form of ALK, phospho-ALK (Y1604), through western blot analysis with lysates of the monolayer-cultured cells. As results, expression levels of these stem cell related molecules in EML4-ALK^+^ cells, H3122 and H2228, were higher than those in EML4-ALK^−^ cells, BEAS-2B and A549 (Figure 1B).

To assess the contribution of EML4-ALK in acquisition of stemness, we treated H3122 and H2228 cells with *ALK*-specific small interfering RNA (*siALK*). Administration of *siALK* reduced the expression of NANOG and OCT4, but not in SOX2, KLF4, and c-MYC (Figure 1C). It was demonstrated that cancer stem cells of NSCLC were characterized by aldehyde dehydrogenase (ALDH) positive population [24, 25]. Consistently, siRNA*-*targeting *ALK* reduced the frequency of ALDH^+^ cells in H3122 cells by one and a half fold and H2228 cells by three fold compared with control *siGFP*-treated cells (Figure 1D). Furthermore, the *siALK*-treated cells formed approximately four fold fewer tumorspheres than the *siGFP*-treated cells (Figure 1E). It was reported that EML4-ALK variant 1 (EAV1) has the highest frequency in patient with EML4-ALK^+^ lung cancer [26, 27]. In addition, H3122 cells expressing EAV1 have higher tumorigenic potential than H2228 cells expressing EAV3 (Figure 1A). Therefore, we evaluated *in vivo* tumorigenicity of EML4-ALK^+^ H3122 cell after transfectoin of *siALK* or *siGFP*. As shown in Figure 1F and 1G, *siALK*-treated cells were significantly less tumorigenic than the *siGFP*-treated cells when transplanted into NOD/SCID mice. These results suggest that EML4-ALK contributes to their stem-like phenotypes and provides tumorigenic advantage to tumor cells.

**Figure 2:**
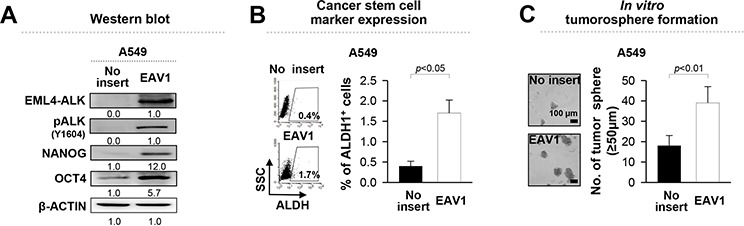
Ectopic expression of *EML4-ALK* enhances the stem-like properties and tumorigenicity of *EML4-ALK* negative cells **A.** A549 cells were transfected with an empty vector or *EML4-ALK variant 1* (*EAV1*) cDNA and the levels of ALK, pALK, NANOG, and OCT4 were analyzed. β-ACTIN was used as an internal loading control. Numbers below blots indicate expression as measured by fold change. **B.** Flow cytometry analysis of the frequency of ALDH1^+^ cells in A549 cells transfected with *EAV1* or empty vector. **C.** Sphere-forming capacity of *EAV1* versus empty vector-transfected A549 cells in a low-density suspension culture. Original magnification, × 40. Error bars represent mean ± SD. Individual data analysis was performed using two-tailed Student's *t*-test.

To demonstrate whether EML4-ALK directly enhances the stem-like phenotypes of NSCLC cells, we retrovirally-transduced EML4-ALK^−^ A549 NSCLC cells with *EML4-ALK* variant 1 (A549/EAV1) or empty vector (A549/no insert), and then characterized their stem-like phenotypes (Figure 2). Compared with A549/no insert cells, ectopic expression of EAV1 increased expression of NANOG and OCT4, and the frequency of ALDH^+^ cells in A549 cells (Figure 2A and 2B). Moreover, overexpression of EAV1 improved the tumorosphere-forming capacity of A549 cells (Figure 2C). Taken together, these data clearly indicate that EML4-ALK confers a stem-like phenotype to NSCLC cells *in vitro* and *in vivo*.

### EML4-ALK inhibitor, crizotinib, diminishes the stem-like properties of EML4-ALK^+^ NSCLC cells

**Figure 3:**
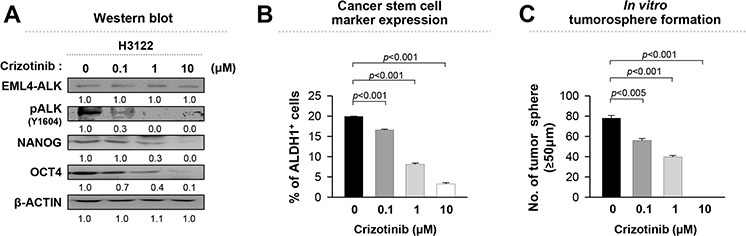
Crizotinib, an ALK inhibitor, reduces the stem-like properties of EML4-ALK positive cells in a dose-dependent manner H3122 cells were treated with crizotinib at the indicated concentration. **A.** ALK, pALK, NANOG, OCT4, and β-ACTIN expression in H3122 cells treated with crizotinib or DMSO (control) for 24 hr was visualized by western blot analysis. Numbers below blots indicate expression as measured by fold change. **B.** Flow cytometry analysis of the frequency of ALDH^+^ cells in H3122 cells treated with crizotinib or DMSO (control) for 24 hr. **C.** Sphere-forming capacity of H3122 cells treated with crizotinib or DMSO (control) in a low-density suspension culture. Original magnification, × 40. Error bars represent mean ± SD. Individual data analysis was performed using two-tailed Student's *t*-test.

Accumulating evidence indicates that ALK activity of EML4-ALK fusion protein mediates tumorigenicity of NSCLC cells via various oncogenic signaling pathways including PI3K/AKT signaling, which is a key regulator of stem-like properties in a cancer stem cell population [17, 28]. On the basis of these findings, we determined whether ALK activity is essential for the stem-like phenotypes of EML4-ALK^+^ NSCLC cells. For this purpose, we treated H3122 cells with crizotinib, an ALK inhibitor, and DMSO as control, and then characterized their stem-like phenotypes. Compared with DMSO-treated H3122 cells, crizotinib-treated cells showed a decrease in expression of NANOG and OCT4 and frequencies of ALDH^+^ cells in a dose-dependent manner (Figure 3A and 3B). The decrease in the expression of stem cell related molecules was not likely to be due to cytotoxicity because more than 80% of cells were alive at the highest dose (1 μM of crizotinib) used in this experiment (Supplementary Figure S1A). The crizotinib-treated cells gradually and consistently lost their sphere-forming capacity in a dose-dependent manner (Figure 3C). Thus, our results indicate that EML4-ALK*-*mediated stem-like phenotypes are dependent on the ALK activity and anti-tumor effects caused by crizotinib treatment at least partially are related with loss of stem-like potentials in EML4-ALK^+^ NSCLC cells.

### Rapamycin decreases stem-like phenotypes in EML4-ALK^+^ NSCLC cells

**Figure 4:**
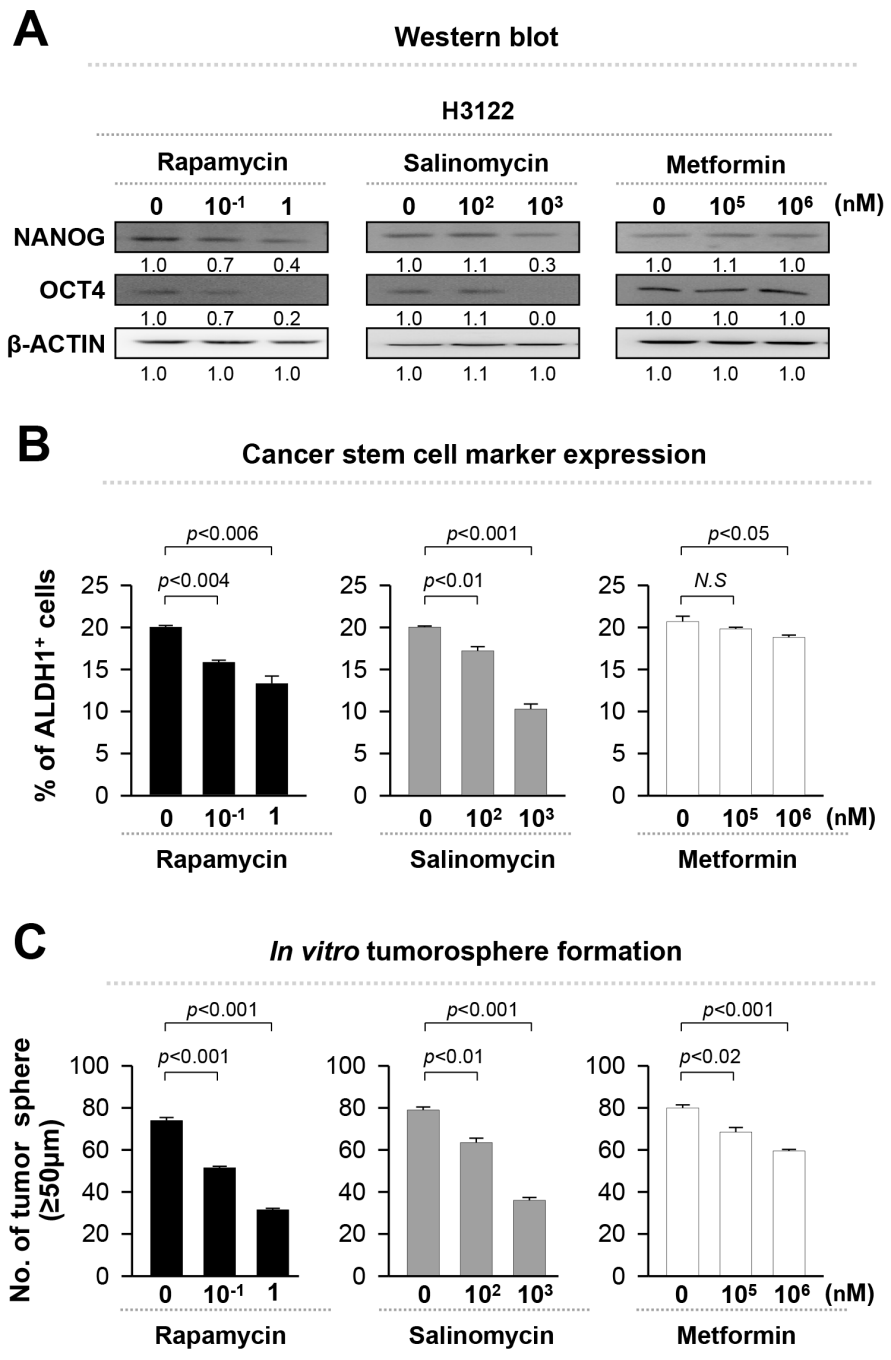
Rapamycin is the most suitable drug that decreases the stem-like properties of EML4-ALK-driven NSCLC cells H3122 cells were treated with rapamycin, salinomycin, and metformin at the indicated concentrations. **A.** H3122 cells were treated with DMSO, rapamycin, salinomycin, or metformin for 24 hr, and the levels of NANOG, OCT4, and β-ACTIN protein were analyzed. Numbers below blots indicate expression as measured by fold change. **B.** Flow cytometry analysis of the frequency of ALDH^+^ cells in H3122 cells treated as in (A). **C.** Quantification of tumorsphere formation with H3122 cells treated with the indicated drugs in a low-density suspension culture. Original magnification, × 40. Error bars represent mean ± SD. Individual data analysis was performed using two-tailed Student's *t*-test.

Although the inhibition of ALK activity by crizotinib treatment can reduce the stem-like properties of EML4-ALK^+^ tumor cells, but its efficacy is limited by variable primary response and acquired resistance. Data from Figure 3 demonstrates that inhibition of stemness effectively reduced tumorigenicity of EML4-ALK^+^ NSCLC cells, suggesting that targeting stemness could be a plausible strategy to treat EML4-ALK^+^ tumor cells. To explore this possibility more directly, we treated EML4-ALK^+^ H3122 cells with well-characterized CSC targeting drugs such as rapamycin, salinomycin, and metformin [21–23, 29]. These drugs showed distinct cytotoxicity over a broad range of doses (Supplementary Figure S1B). Among them, rapamycin was the most effective in decreasing the expression level of stem cell related molecules and the CSC marker, and in inhibiting the tumorosphere formation in the nano-molar range of sub-lethal doses (Figure 4A, 4B and 4C). Hence, our data demonstrates that rapamycin significantly reduces the stem-like properties of EML4-ALK^+^ cells.

### Co-treatment with rapamycin enhances the anti-tumor effects of crizotinib on EML4-ALK^+^ NSCLC cells

**Figure 5:**
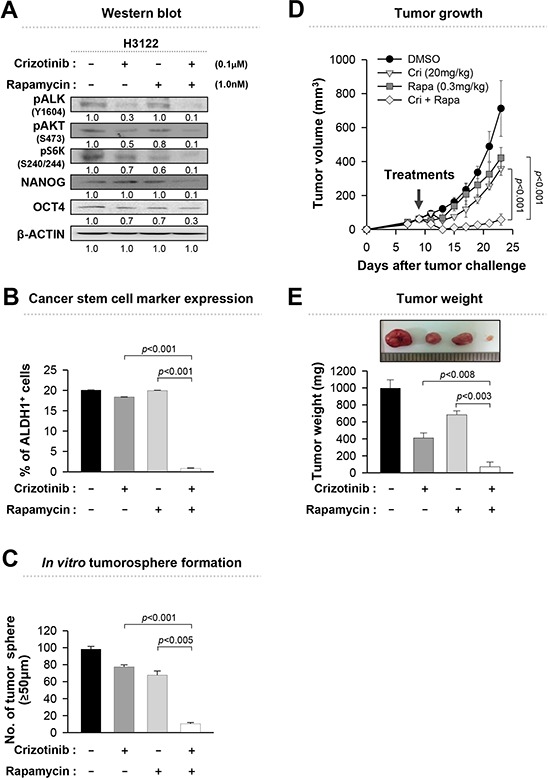
Inhibition of EML4-ALK-mediated stem-like properties enhances the anti-tumor effect **A.** Western blot analysis using antibodies specific to the proteins in lysates from H3122 cells that treated with the indicated drugs for 24 hr. Numbers below blots indicate expression as measured by fold change. **B.** Flow cytometry analysis of the frequency of ALDH^+^ cells in H3122 cells treated as in (A). **C.** Quantification of tumorsphere-formation with H3122 cells treated with the indicated drugs in a low-density suspension culture. Original magnification, × 40. **D.** Tumor growth in mice inoculated with H3122 cells. Nude mice were inoculated subcutaneously with 1 × 10^6^ cells/mouse. Nine days following tumor challenge, the CH containing the indicated drug or both drugs was injected intratumorally. **E.** Tumor weight in mice at 23 days after the challenge. Error bars represent mean ± SD. Individual data analysis was performed using two-tailed Student's *t*-test.

Stem-like properties are reported to be involved in tumorigenicity and survival of recurrent tumor after the conventional cancer therapy [15, 19, 20]. As shown in Figure 4, rapamycin appeared to decrease the stem-like potential of the EML4-ALK^+^ NSCLC cells. On the basis of those observations, we tested whether co-treatment with stemness-targeting drugs such as rapamycin could enhance anti-tumor effects of crizotinib. To verify it, we treated EML4-ALK^+^ H3122 cells with indicated concentrations of crizotinib and rapamycin, either alone or in combination as shown in Figure 5A. Co-treatment with crizotinib and rapamycin synergistically decreased the expression level of the stemness factor such as NANOG and OCT4 along with the frequency of ALDH^+^ cells compared to each single treatment (Figure 5A and 5B). Furthermore, compared to single-agent treatments, co-treatment resulted in formation of almost ten fold fewer tumorospheres (Figure 5C).

To evaluate *in vivo* anti-tumor effects, thermo-sensitive chitosan hydrogels containing cancer therapeutic agents have been previously used for sustained release of drugs at local tumor sites [30]. To assess the therapeutic value of co-administration of crizotinib and rapamycin, H3122 cells were inoculated into nude mice, and then 9 days later, chitosan hydrogels containing crizotinib, rapamycin, or both were administered intratumorally. Although treatments with each single-agent elicited a profound therapeutic effect, co-treatment was much more effective at delaying the growth of the EML-ALK^+^ xenograft as shown in Figure 5D. Also co-treatment effectively reduced the weight of the tumor (Figure 5E). Taken together, these data clearly demonstrate that co-treatment with crizotinib and rapamycin is superior in potency than treatment with either agent alone in EML4-ALK^+^ NSCLC cells.

### Rapamycin sensitizes crizotinib-resistant H3122 CR1 cells to ALK inhibition

**Figure 6:**
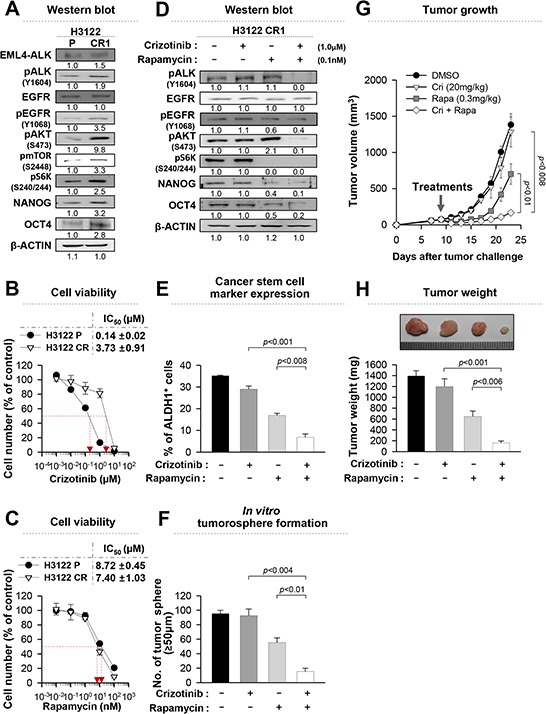
Rapamycin can effectively reduce the stem-like properties of crizotinib-resistant cells **A.** Western blot analysis using antibodies specific to the proteins in lysates from H3122 cells or H3122 CR1 cells. Numbers below blots indicate expression as measured by fold change. **B.** and **C.** Dose-response curves for the viability of control H3122 cells and H3122 CR1 cells treated with crizotinib or rapamycin for 72 hr. **D.** Western blot analysis using antibodies specific to the proteins in lysates from H3122 CR1 cells that treated with the indicated drugs for 24 hr. Numbers below blots indicate expression as measured by fold change. **E.** Flow cytometry analysis of the frequency of ALDH^+^ cells in H3122 CR1 cells treated as in (D). **F.** Quantification of tumorsphere-formation with H3122 CR1 cells treated with the indicated drugs in a low-density suspension culture. Original magnification, × 40. **G.** Tumor growth in mice inoculated with H3122 CR1 cells. Nude mice were inoculated subcutaneously with 1 × 10^6^ cells/mouse. Nine days following tumor challenge, the CH containing the indicated drug or both drugs was injected intratumorally. **H.** Tumor weight in mice at 23 days after challenge. Error bars represent mean ± SD. Individual data analysis was performed using two-tailed Student's *t*-test.

Previously, we established H3122 CR1 cells that were resistant to crizotinib by exposure to increasing concentrations of crizotinib through an *in vitro* long-term culture. H3122 CR1 cells have the L1196M of *ALK* mutation, and display ligand-dependent EGFR activation by up-regulating amphiregulin and EGF [11]. EGFR signaling is one of the major upstream regulators of mTOR, and its activation induces resistance to crizotinib [9] as well as cancer stemness [31, 32]. In agreement with this finding, the EGFR-mTOR signaling pathway was activated in crizotinib-resistant H3122 CR1 cells compared to crizotinib-sensitive (parental) H3122 cells as shown in Figure 6A. The expression levels of NANOG and OCT4 were also higher in the H3122 CR1 cells than in the H3122 cells. On the basis of these observations, we inhibited mTOR signaling by using repamycin, and determined the effects on crizotinib sensitivity in H3122 CR1 cells. As expected, H3122 CR1 cells were sensitive to rapamycin treatment while they were resistant to crizotinib treatment (Figure 6B and 6C). Combined treatment synergistically reduced the expression levels of the stemness factors and the frequency of ALDH^+^ cells compared to single-agent treatment (Figure 6D and 6E). Moreover, co-treatment led to formation of almost four to six fold fewer tumorospheres compared to single-agent treatment (Figure 6F). These findings indicate that EGFR-mTOR pathway has a role in maintaining stem-like phenotypes and downstream signaling in the presence of continuous ALK inhibition.

These findings prompted us to further investigate *in vivo* anti-tumor effects of rapamycin alone or in combination with crizotinib on H3122 CR1 cells. Interestingly, co-treatment was more effective at delaying the growth rate of the H3122 CR1 xenograft tumor as shown in Figure 6G, although treatment with rapamycin alone also elicited a profound therapeutic effect. In contrast, single treatment with crizotinib failed to retard the growth of the xenograft tumor at the dose used in the experiment. In addition, co-treatment resulted in the lowest tumor weight for the established tumors (Figure 6H). Taken together, these data suggest that the ALK and mTOR inhibitor combination may provide an attractive treatment strategy for ALK TKI-resistant states.

## DISCUSSION

In this study, we examined, for the first time, the role of EML4-ALK in stem-like properties of NSCLC tumor cells, which was validated both *in vitro* and *in vivo*. EML4-ALK up-regulated the expression of NANOG and OCT4 proteins, which is dependent on its ALK enzymatic activity. Consistently, ALK inhibition with crizotinib reversed the stem-like phenotypes of EML4-ALK^+^ tumor cells, suggesting ALK signaling is a central molecular axis in the stem-like phenotype of EML4-ALK^+^ tumor cells. Of note, it is a remarkable finding that EML4-ALK induces a stem-like phenotype in NSCLC cells. This may not be entirely surprising since EML-ALK activates various downstream signaling molecules including ERK, STAT3, and AKT, which are the key elements in inducing and maintaining stem-like properties for both normal stem cells and cancer stem cells [17, 18, 28, 29, 33]. Nonetheless, little is known about the molecular mechanisms by which ALK functions. A further study is needed to elucidate the role of ALK signaling in the development of the stem-like phenotype of NSCLC cells.

The mammalian target of rapamycin (mTOR), a serine/threonine kinase existing in two functionally distinct complexes (mTORC1 and mTORC2), is commonly up-regulated in various human cancers including NSCLC [34–37]. Besides, there have been reports suggesting that the AKT-mTOR axis is associated with the maintaining cancer stemness. Cancer stem cells also show preferential sensitivity to inhibition of the pathway when compared to normal stem cells [38, 39]. Importantly, in accordance with the previous findings, the mTOR inhibitor rapamycin effectively suppressed the EML4-ALK-induced stem-like characteristics at nano-molar doses. As shown in Supplementary Figure S2A, even at 1nM dose, rapamycin, was potent enough to decrease the phosphorylation of mTOR and its target S6K by over two fold without affecting phosphoylation of ALK. To find out clue to the function of an mTOR inhibitor that decreases the stem-like phenotypes of EML4-ALK^+^ cells, we further investigated the relationship between the EML4-ALK-induced stem-like phenotypes and AKT-mTOR signaling in EML4-ALK^+^ H3122 cells treated with *siGFP* or *siALK* (Supplementary Figure S2B). Interestingly, *siALK* treatment drastically reduced the phosphorylation of AKT on threonine 308 (T308), which is an upstream regulator of mTOR, and in turn dampened the phosphorylation of mTOR and its target S6K by over three fold compared to control *siGFP* treatment. Conversely, retroviral transduction of EML4-ALK^−^ A549 NSCLC cells with *EML4-ALK* variant 1 led to activation of both AKT and mTOR molecules (data not shown). Consistently, previous studies by other groups also have reported that EML4-ALK activates various downstream signaling molecules including AKT and mTOR [40]. Moreover, aberrant mTOR activation led to the increase of NANOG and OCT4 expression [33]. As shown in Figure 4A, we also have found that the mTOR inhibitor rapamycin reduced expression of NANOG and OCT4 in EAV1-exressiong H3122 cells. Taken together, those data clearily demonstrate mTOR signaling is essential in EML4-ALK-mediated stem-like properties although an exact mechanism how mTOR signaling can regulate the expression of NANOG and OCT4 in EML4-ALK^+^ NSCLC cells still remains elusive.

It has been documented that targeting stemness of cancer can potentiate the effects of chemotherapy or radiotherapy [14, 41–43]. Similarly, we propose that treatment with stemness-targeting agent rapamycin could sensitize EML4-ALK^+^ NSCLC cells to conventional cancer drugs including ALK inhibitors. In this study, we clearly demonstrated that mTOR inhibition renders EML4-ALK^+^ NSCLC cells more vulnerable to crizotinib-mediated apoptotic death, and this inhibition even re-sensitizes acquired crizotinib-resistant NSCLC cells, H3122CR1, to crizotinib. Similar results were also observed in SNU-2535 cells previously derived from a crizotinib-resistant patient who harbored the G1269 mutation in the ALK domain [11]. One plausible explanation for the synergistic anti-tumor effects is that rapamycin and crizotinib share common AKT-mTOR axis and its down-stream target molecules, NANOG and OCT4, as these are considered to be essential for tumor maintenance in many malignancies [28, 44, 45].

Recently, we reported about the ligand-dependent EGFR activation in both H3122 CR1 cells and SNU-2535, a lung cancer cell line derived from a patient who had developed acquired resistance to crizotinib [11]. It is noteworthy the crizotinib-resistant H3122 CR1 cells had more phosphorylated EGFR when compared with H3122 cells (Supplementary Figure S3A). Consistently, the level of EGFR ligands such as amphiregulin and EGF is significantly higher in H3122 CR cells than in H3122 cells [11]. The level of pEGFR in H3122 CR cells substantially decreased by adding rapamycin but not crizotinib as shown in Figure 6 and Supplementary Figure S3B. It has been reported that an aberrant mTOR signal cascade increases the expression of various growth factors including EGFR ligands [46]. Those observations suggests mTOR signaling could trigger the activation of EGFR by up-regulating EGFR ligands in in EML4-ALK^+^ NSCLC cells. This possibility need to be elucidated. Nevertheless, however, it is clear that a bypass mechanism via activation of EGFR-AKT-mTOR signaling could be one of the causes of crizotinib resistance in H3122 CR1 cells, and rapamycin definitely abrogated the acquired resistance due to EGFR activation by targeting its downstream signaling molecule, mTOR. Since other bypass pathways of acquired ALK resistance, via KIT, KRAS, or IGF-1R, could also be converged on AKT-mTOR axis, rapamycin could be effective in sensitizing these ALK resistant cells caused by the bypass signalings. Collectively, our results suggest that targeting of mTOR can be helpful in reversing the acquired crizotinib resistance.

Taken together, our findings clearly provide new insights suggesting that stem-like properties can be a pivotal driver of EML4-ALK-mediated tumorigenicity of NSCLC cells and inhibition of mTOR signaling can be a potential strategy to clinically control intractable stem-like phenotypes of EML4-ALK^+^ NSCLC.

## MATERIALS AND METHODS

### Mice

Six- to eight-week-old female nude and NOD/SCID mice (Raon Bio Inc., Korea) were maintained and handled under the protocol approved by the Korea University Institutional Animal Care and Use Committee (KUIACUC-2014-32). All animal procedures were conducted in accordance with recommendations for the proper care and use of laboratory animals.

### DNA constructs

To generate pMSCV/EML4-ALK variant 1 construct, the DNA fragment encoding EML4-ALK variant 1 was amplified from pBabe-EML4-ALK variant 1 [1] using a set of primers : forward : 5′- GCGAATTCGGTTTCGCCGGCAGTCT - 3′, reverse : 5′ - GCGAATTCAGACTGCCGGCGAAACC - 3′. The PCR product was continuously cloned into *EcoRI* restriction sites of the pMSCV retroviral vector (Clontech, Korea). In the experiment, plasmid integrity was confirmed by DNA sequencing.

### Reagents and cell lines

Crizotinib, rapamycin, salinomycin, metformin and puromycin were purchased from Sigma (St.Louis, MO, USA). Each compound was dissolved in DMSO for cell culture experiments. But metformin was dissolved in PBS. Human NCI-H3122 (parental) cells were provided by Pasi A. Janne (Dana-Farber Cancer Institute, Boston, MA, USA). NCI-H2228 and A549 cells were purchased from the American Type Culture Collection (ATTC; Manassas, VA, USA). SNU-2535 cells were established at the Korean Cell Bank using pleural effusion in a patient who had developed acquired resistance to crizotinib. H3122 crizotinib-resistant (H3122 CR1) cells that were generated by an increasing dose of crizotinib [11]. H3122, H3122 CR1, SNU-2535, and A549 cells were cultured in RPMI1640 supplemented with 10% FBS. All cells were cultured at 37°C in 5% CO_2_ atmosphere. The A549 EAV1 (EML4-ALK variant 1) cell line was produced by retroviral transduction of A549 cells with pMSCV/EML4-ALK variant 1 using pMSCV vector-Phoenix packaging cell line system followed by puromycin (1 μg/ml) selection.

### siRNA constructs

Synthetic *siGFP*, *siALK* had the following sequences : GFP, 5′-GCAUCAAGGUGAACUUCAA-3′(sense), 5′ - UUGAAGUUCACCUUGAUCGC - 3′(antisense); ALK, 5′ - CCGCUUUGCCGAUAGAAUA - 3′(sense), 5′ - UAUUCUAUCGGCAAAGCGG - 3′(antisense); and they were obtained commercially (Bioneer Inc., Korea). siRNA was delivered *in vitro* into 6-well plates at a dose of 200 pmol per well using Lipofectamine 2000 (Invitrogen, Korea).

### Flow cytometry analysis

H3122, H3122 CR1, and A549 cells were harvested by trypsinization and stained using the Aldefluor^®^ stem cell detection kit (Stem Cell Technologies), as previously described [24]. Briefly, cultured cells were incubated in ALDEFLUOR assay buffer containing the ALDH protein substrate (BAAA, 1 μmol/1 L per 1 × 10^6^ cells) for 45 min at 37°C. In each experiment, a sample of cells was stained by the addition of 40 mmol/L of diethylaminobenzaldehyde (DEAB), a specific ALDH inhibitor during the incubation, as negative control. All data analysis was conducted on a FACSCalibur flow cytometer (BD Biosciences, San Jose, CA, USA) with CellQuest Pro software. Also, all data were normalized to the control group and presented as mean ± standard deviation.

### Western blot

5 × 10^5^ cells were rinsed twice with PBS and lysis buffer (50 mM Tris, 150 mM NaCl, 1 mM phenylmethylsulfonyl fluoride, 0.1% SDS, 1% NP-40, 0.5 mM EDTA) was added. Protein concentration determination and immunoblotting were conducted as described previously [28]. Primary antibodies against ALK, pALK (Y1604), c-MYC, EGFR, pEGFR (Y1068), AKT, pAKT (T308), pAKT (S473), mTORC1, pmTORC1 (S2448), S6K, pS6K (S240/244) (Cell Signaling Technology, MA, USA); SOX2, OCT4, and KLF4 (Santa Cruz Biotechnology, Dallas, TX, USA); NANOG (Abnova, CA, USA) were used at 1:1000–1:3000 dilution. The β-ACTIN antibody (1:10000 dilution) was purchased from Sigma. Immune-reactive bands were visualized by enhanced chemiluminescence imaging (Elpis Biotech, Korea).

### Tumorsphere-forming assay

Tumorsphere formation was measured as previously described [28]. H3122, H3122 CR1, or A549 cells were plated at 1 × 10^4^ cells per well in 24-well, super-low adherence vessels (Corning, USA) containing serum-free DMEM-F12 (Thermo Scientific, USA) supplemented with 1X B27, basic fibroblast growth factor (20 ng/ml), and epidermal growth factor (20 ng/ml). Crizotinib, Rapamycin, Salinomycin and Metformin were added to the sphere culture at suboptimal doses. Tumorspheres more than 50 μm in diameter were counted under a microscope.

### Tumorigenicity assay

H3122 cells were transfected with *siGFP* and *siALK* twice using Lipofectamine 2000 at a 24-hr interval. Twenty-four hours after secondary transfection, viable cells were harvested by trypsinization and then they were washed and resuspended in Opti-MEM. NOD/SCID mice were subcutaneously injected with 10^4^ or 10^5^ cells. Tumor formation was monitored at least 3 times per week and for 27 days after tumor injection. After 20 days, tumor tissue was excised and weighed.

### Tumor treatment experiments

For *in vivo* treatment experiments, nude mice were inoculated subcutaneously with 10^6^ H3122 or H3122 CR1 tumor cells per mouse. Before starting treatment, mice (*n* = 5 per group) were randomized to: a) DMSO (control), b) crizotinib only (20 mg/kg), c) rapamycin only (0.3 mg/kg), or d) combination treatment, and they were monitored regularly for 23 days. In addition, tumor dimensions (length and width) were measured 3 times per week with calipers, and tumor volume was calculated as length (mm) × width^2^ (mm^2^) × 0.52. For drug treatment, CH (chitosan hydrogel) containing the drug or DMSO was injected intratumorally. CH was prepared as described previously [30].

### Statistical analysis

All data are representative of at least three separate experiments. Individual data analysis was performed using two-tailed Student's *t*-test. Statistical analysis was performed with SPSS version 12.0 software (SPSS Inc., Chicago, IL). In all cases, statistical significance was established at *P* value < 0.05.

## SUPPLEMENTARY FIGURES


