# Supplementary material for: Targeting stemness is an effective strategy to control EML4-ALK+ non-small cell lung cancer cells
Source: Oncotarget. 2015 Oct 22;6(37):40255–67. doi: 10.18632/oncotarget.5434 (PMC4741893; doi:10.18632/oncotarget.5434)
Supplement: Supplementary file 1 [file oncotarget-06-40255-s001.pdf]

SUPPLEMENTARY FIGURES

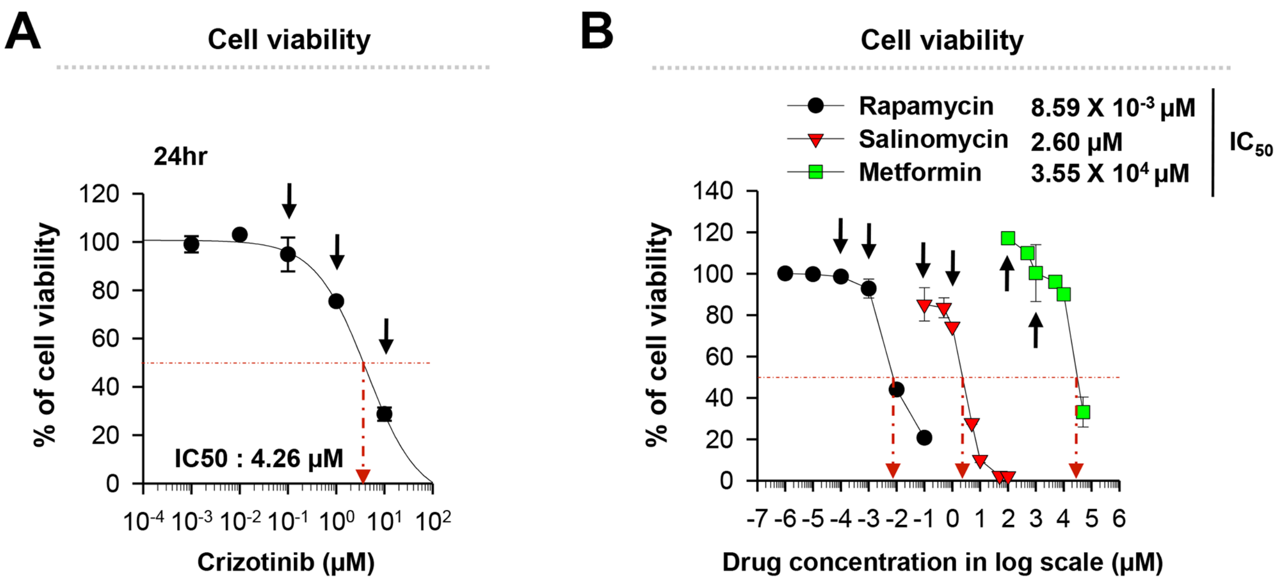

Supplementary Figure S1: Cell survival assay of H3122 following treatment with crizotinib or inhibitors of cancer stem-like cells. A. H3122 cells were seeded in 96-well plates and treated with crizotinib for 24 hr. Cell survival was analyzed using the MTT cell viability assay. **B.** Dose-response curves for the viability of H3122 cells treated with rapamycin (black), salinomycin (red), or metformin (green) for 72 hr. Error bars represent mean ± SD.

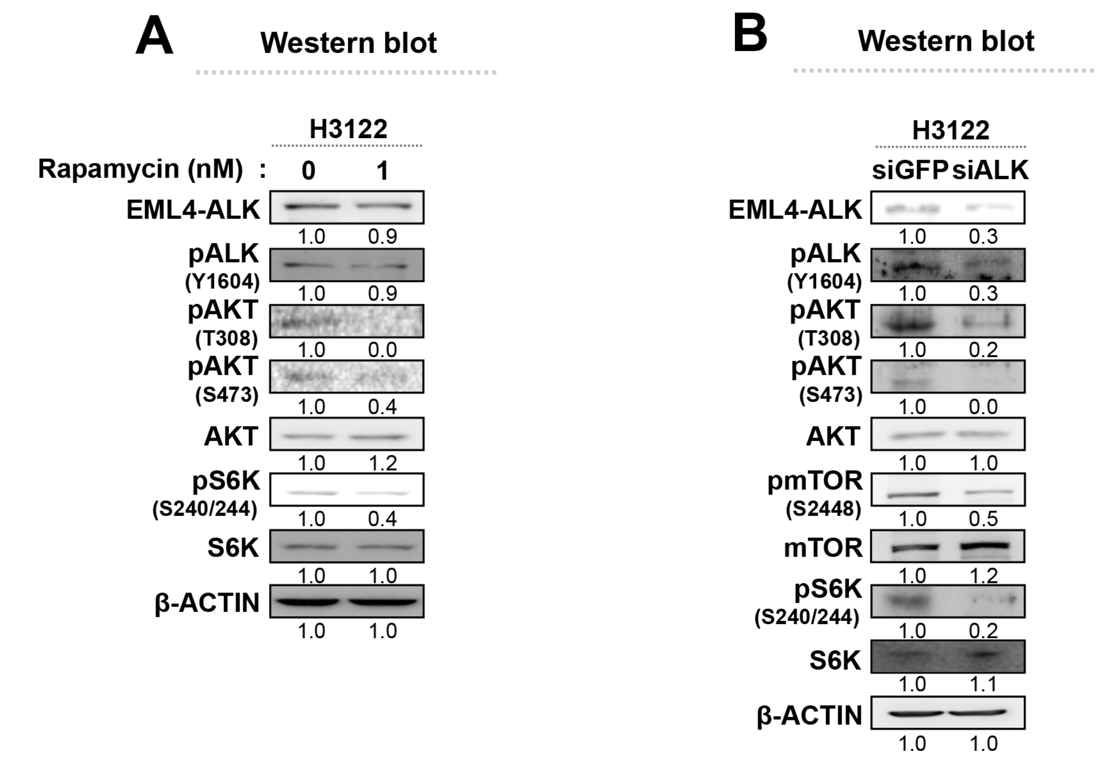

Supplementary Figure S2: EML4-ALK triggers the AKT-mTOR pathway in H3122 cells. Numbers below blots indicate expression as measured by fold change. **A.** Western blot analysis using antibodies specific to the proteins in lysates from H3122 cells. **B.** H3122 cells were treated with *siGFP* (control) or *siALK* and the levels of ALK , pALK, pAKT (T308), pAKT (S473), AKT, pmTOR (S2448), mTOR, pS6K (S240/244), and S6K protein were analyzed. β-ACTIN was used as an internal loading control.

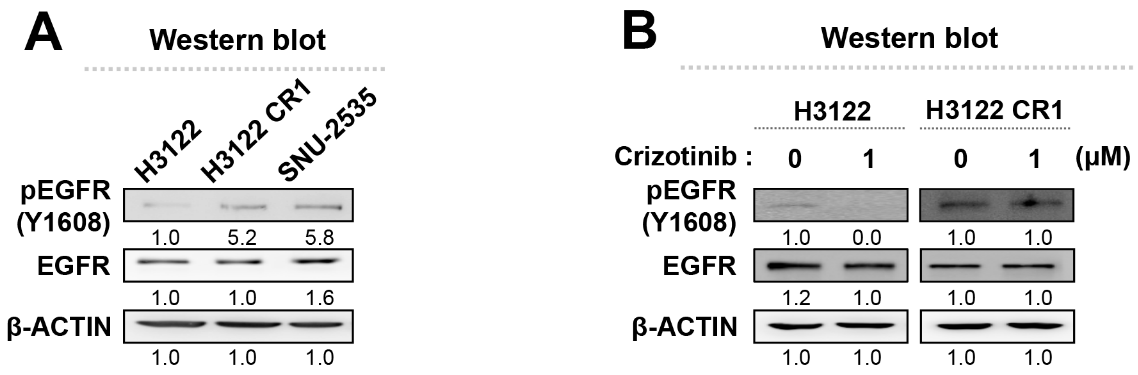

Supplementary Figure S3: Crizotinib-resistant cells showed an increased level of phosphorylated EGFR compared with parental cells. Numbers below blots indicate expression as measured by fold change. **A.** Western blot analysis using antibodies specific to the proteins in lysates from H3122, H3122 CR1, and SNU-2535 cells. **B.** H3122 and H3122 CR1 cells were treated with DMSO (control) or crizotinib (1 μM) for 24 hr, and the levels of pEGFR (Y1068) and EGFR protein were analyzed. β-ACTIN was used as an internal loading control.
